# Comparison of nedaplatin-based versus cisplatin-based chemotherapy for advanced non-small cell lung cancer among East Asian populations: A meta-analysis

**DOI:** 10.1038/srep10516

**Published:** 2015-05-21

**Authors:** Yun Liu, Shaorong Yu, Siwen Liu, Haixia Cao, Rong Ma, Jianzhong Wu, Jifeng Feng

**Affiliations:** 1 Department of Chemotherapy, the Affiliated Jiangsu Cancer Hospital, Nanjing Medical University; 2 Research Center of Clinical Oncology, the Affiliated Jiangsu Cancer Hospital, Nanjing Medical University, Nanjing, Jiangsu 210009, China

## Abstract

Whether nedaplatin and cisplatin are equally effective for advanced non-small cell lung cancer (NSCLC) remains uncertain. Therefore, we performed a meta-analysis of trials to compare nedaplatin-based chemotherapy with cisplatin-based chemotherapy. We conducted a literature search to identify trials that had investigated the substitution of nedaplatin for cisplatin in the treatment of advanced NSCLC. Fourteen randomized controlled trials were included. We found equivalent overall response, overall survival, and survival probability (0.5-year, 1-year). Considering the toxicity profiles, nausea and vomiting were common in the cisplatin group (OR = 0.28, 95% CI = 0.20–0.40, P < 0.001), whereas severe thrombocytopenia was common in the nedaplatin group (OR = 1.68, 95% CI = 1.18–2.40, P = 0.005). A subgroup analysis of grades 1–4 nephrotoxicity showed that cisplatin-based chemotherapy resulted in more renal toxicity (OR = 0.40, 95% CI = 0.24–0.68, P = 0.001). No significant heterogeneity and publication bias were observed. Cumulative analysis found a stable time-dependent trend. Consistent results stratified by age, regimen, and country were observed. Cisplatin-based chemotherapy was associated with non-inferior antitumor efficacy compared with nedaplatin-based therapy. Therefore, the toxicity profile might play an important role in choosing between cisplatin-based or nedaplatin-based regimens.

Lung cancer is the leading cause of cancer-related deaths in many countries, and non-small cell lung cancer (NSCLC) accounts for approximately 85%–90% of lung cancer diagnoses. At the initial time of diagnosis, only 25%–30% of patients with NSCLC are able to seek surgery^1^,^2^,^3^. For the 30% of patients with local advanced or metastatic disease, the main treatment is limited to supportive care and chemotherapy, and surgery is inapplicable^4^,^5^. Despite the advances in chemotherapy, local and distant control remain suboptimal, and the majority of patients continue to die from distant metastases. Platinum-based chemotherapies are still the standard regimens considered as the first-line chemotherapy for advanced NSCLC by the American Society of Clinical Oncology and National Comprehensive Cancer Net^5^,^6^.

Cisplatin (DDP) is the first generation of platinum drugs, which has become the basic drug for treating advanced solid tumors, such as NSCLC^7^. However, its clinical application is limited because of severe gastrointestinal responses, renal toxicity, and neurotoxicity^8^,^9^,^10^,^11^. To avoid DDP-induced toxicities, nedaplatin (NDP), an analog of DDP, was introduced into clinical trials in Japan in 1990^12^. This new anti-tumor drug derived from DDP, which has similar action mechanisms to DDP, revealed stronger anti-tumor effects and lower toxic responses in animal research^13^. A two-drug combination consisting of NDP plus a new chemotherapy agent has been frequently used in clinical practice, as well as in clinical trials, particularly in China and Japan. Although many randomized controlled trials were performed to compare NDP-based chemotherapy with DDP-based chemotherapy, the results remain uncertain^14^,^15^,^16^,^17^,^18^,^19^,^20^,^21^,^22^,^23^,^24^,^25^,^26^,^27^.

Therefore, we performed a meta-analysis to compare the effects and toxicities between NDP-based and DDP-based regimens as the first-line treatment of patients with advanced NSCLC.

## Results

### Eligible studies

**Figure 1:**
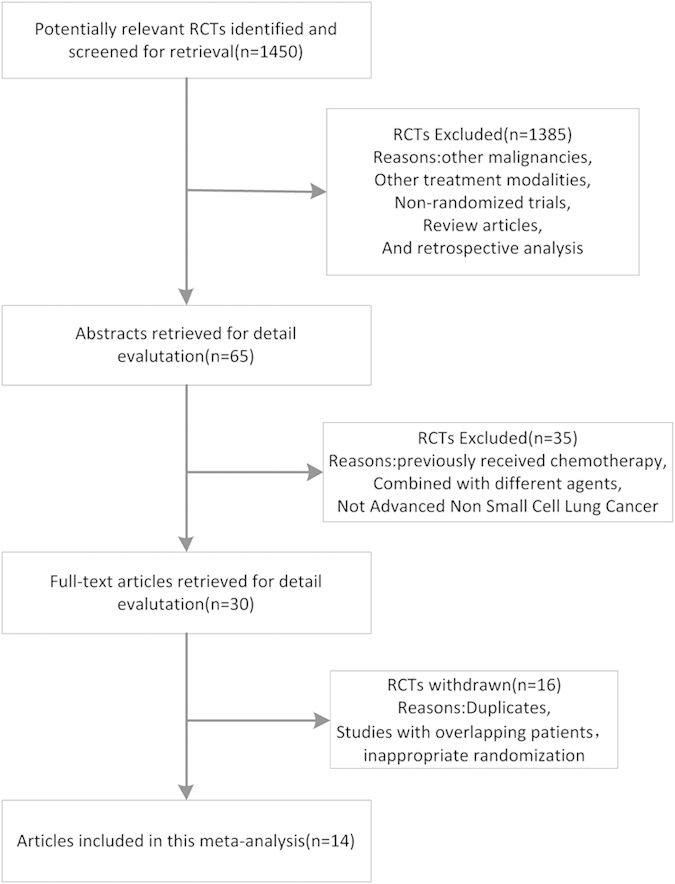
A flow chart showing the progress of trials through the review. RCT, randomized controlled trials.

A flow chart of our study is shown in Fig. 1. Fourteen randomized trials that satisfied the inclusion criteria were identified with a total of 634 patients in the NDP-based arm and 608 patients in the DDP-based arm (Table 1). No significant differences were found in the baselines between the NDP-based arm and DDP-based arm in these studies. The quality of the 14 trials was assessed using the three-question instrument reported by Jadad *et al.*^28^. The quality scores are also listed in Table 1. Given the particularity of chemotherapy, none of the trials were double-blinded. All trials reported withdrawals or dropouts, and a statement regarding randomization was included in all. Ten out of the 14 trials described the detailed methods of randomization^14^,^15^,^16^,^17^,^18^,^20^,^23^,^24^,^25^,^26^, which were scored 2, and the others were scored 1.

### Overall response

**Figure 2:**
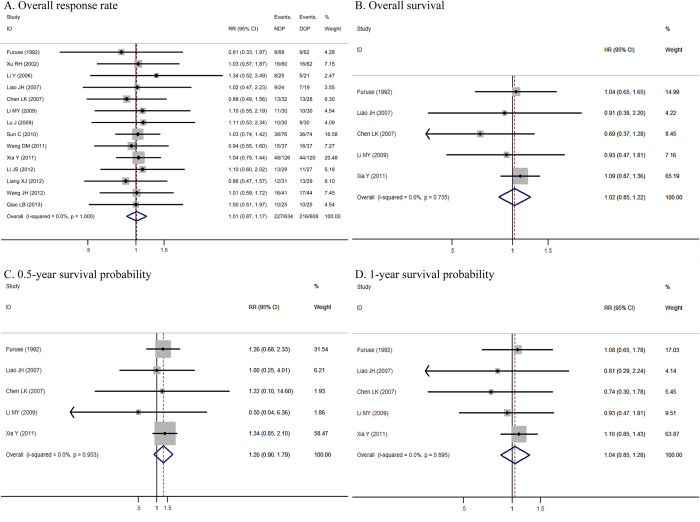
Forest plots estimating the overall response rate (**A**) overall survival (**B**) 0.5-year survival probability (**C**) and 1-year survival probability (**D**) in the comparison of nedaplatin-based versus cisplatin-based chemotherapy.

All the 14 studies demonstrated an overall response. Overall response was defined as complete response plus partial response. The overall response of the NDP-based arm ranged from 11.8% to 50.0%, whereas that of the DDP-based arm ranged from 14.5% to 48.6%. This meta-analysis showed an equivalent overall response between NDP-based and DDP-based chemotherapy (35.8% vs. 35.5%; RR = 1.01, 95% CI = 0.87–1.17, P = 0.89, I^2^ = 0.0%). The details are illustrated in Fig. 2A.

### Overall survival (OS) and survival probability

Median OS was demonstrated in six studies, which ranged from 8.9 to 14.3 and 9.1 to 13.0 months for NDP-based and DDP-based chemotherapy, respectively. Survival analyses were carried out based on intention-to-treat analysis in five trials. The pooled hazard ratio (HR) for OS of five studies showed that DDP-based chemotherapy was associated with only a 2% improvement in OS as compared with NDP-based chemotherapy, and this difference was not statistically significant (HR = 1.02, 95% CI = 0.85–1.22, P = 0.82, I^2^ = 0%) (Fig. 2B). These five studies published Kaplan–Meier curves of OS. Meta-analysis of 0.5- and 1-year survival probability found no significant differences between the two arms (0.5-year RR = 1.27, 95% CI = 0.90–1.79, P = 0.18, I^2^ = 0%; 1-year RR = 1.04, 95% CI = 0.85–1.28, P = 0.69, I^2^ = 0%) (Fig. 2C,D).

### Toxicity

All trials provided toxicity profiles in a per-patient manner, except one that reported toxicities in a per-treatment cycle manner, so it was excluded from the analyses^18^. Table 2 presents a the summary of toxicity results. DDP-based chemotherapy led to more common grade 3 or 4 nausea and vomiting (OR = 0.28, 95% CI = 0.20–0.40, P < 0.001), whereas NDP-based chemotherapy resulted in the development of grade 3 or 4 thrombocytopenia (OR = 1.68, 95% CI = 1.18–2.40, P = 0.005). The risk of grade 3 or 4 anemia, neutropenia, neurotoxicity, and nephrotoxicity was almost comparable between the two arms (OR = 1.01, 95% CI = 0.67–1.53, P = 0.95; OR = 1.07, 95% CI = 0.81–1.40, P = 0.65; OR = 0.62; 95% CI = 0.16–2.41, P = 0.49; and OR = 0.31, 95% CI = 0.09–1.06, P = 0.06, respectively). Considering the low incidence of grades 3–4 nephrotoxicity, we compared grade 1–4 nephrotoxicity in eight trials^14^,^15^,^16^,^17^,^20^,^23^,^24^,^25^. The results showed that DDP-based chemotherapy significantly developed more renal toxicity than NDP-based chemotherapy (OR = 0.40, 95% CI = 0.24–0.68, P = 0.001). To investigate the difference in neutropenia between the two groups, subgroup analysis was conducted compare the grades of neutropenia (grades 1–4) in 11 trials^14^,^15^,^16^,^17^,^20^,^21^,^22^,^23^,^24^,^25^,^26^, and similar results were obtained (OR = 1.07, 95% CI = 0.80–1.42, P = 0.66).

### Heterogeneity, regression analysis, and publication bias assessment

**Figure 3:**
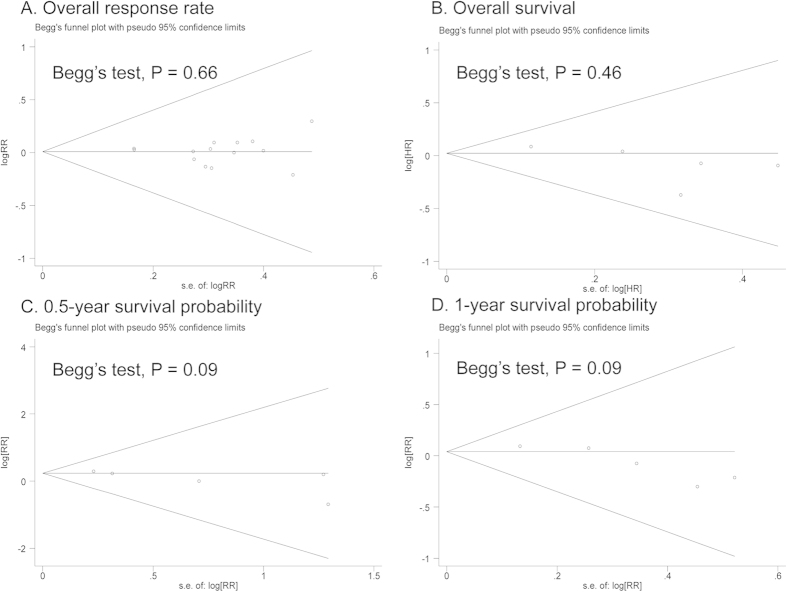
Begg’s funnel plots.

No significant heterogeneity was found for all analyses (I^2^ < 50%, P > 0.05). When a fixed-effects model was changed to a random-effects model for all comparisons, the test of significance obtained the same conclusion. Meta-regression analysis further found that the patient median age (patient median age ≤60 or >60) of each group was not a major contributor to between-study heterogeneity (P = 0.79), without sufficient information for other string variables. 1-year survival probability showed borderline publication bias via Egger’s test (P = 0.03), but no publication bias was determined by Begg’s test (P = 0.09). Moreover, no publication bias was observed for other results, with a symmetrical appearance on funnel plot analysis, in which P ranging from 0.09 to 0.66 was given by Begg’s test, and P ranging from 0.10 to 0.92 was given by Egger’s test (Fig. 3).

### Subgroup analysis

Although no significant heterogeneity was observed in all the comparisons, we probed into detailed results in subgroup analyses stratified by patient median age (patient median age ≤60 or >60), chemotherapy regimen (combined with VDS, NVB, GEM, PTX, or TXT), and country (Japan or China). All subgroup results were quite consistent with the overall results. No significant heterogeneity was found in all subgroup analyses. The results are summarized in Table 3.

### Cumulative meta-analysis

**Figure 4:**
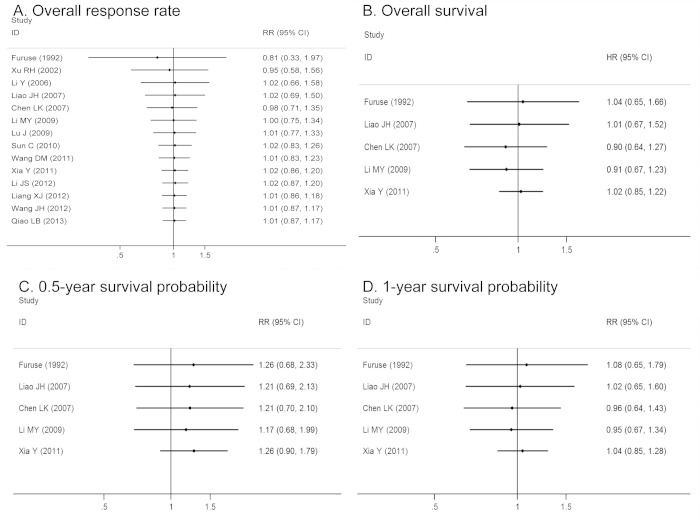
Cumulative meta-analysis to sort out the time-tendency of outcomes.

The time span of the available studies was considerable (from 1992 to 2013), so a cumulative meta-analysis was performed to identify the time-tendency of outcomes by successively adding studies to the given result. For overall response, OS, and survival probability, this cumulative meta-analysis consistently and steadily showed equivalent effects of DDP-based chemotherapy versus NDP-based chemotherapy. Several initial studies were pooled, which showed a narrow range of 95% CI (Fig. 4).

## Discussion

In the present meta-analysis, we demonstrated that the two chemotherapy regimens produced equivalent efficacy as first-line treatment for advanced NSCLC. The cumulative meta-analysis suggested that the findings were robust with time.

As an important measure of anti-tumor efficacy, overall response demonstrated that NDP-based chemotherapy was equivalent to DDP-based chemotherapy, which had a high degree of consistency with each included study. As the most meaningful measure of treatment effect for cancer, the pooled HR showed comparable OS of these two therapies (HR = 1.02, 95% CI = 0.85–1.22), which slightly favored DDP-based therapy. Furthermore, equivalent 0.5-year and 1-year survival probabilities, which were consistent with the case in each included study, reinforced the comparable efficacy of the two therapies. In subgroups stratified by potential confounders (regimen, age, and country), equivalence in efficacy was found and was quite consistent with the overall results.

Physicians should carefully interpret these results when they apply them in clinical practice because the toxicity profiles were quite different between these two regimens. Grades 3 and 4 nausea and vomiting were more common in the DDP-based therapy group, whereas severe thrombocytopenia was more common in the NDP-based therapy group. Considering the lower rates of grades 3 and 4 nephrotoxicity, a subgroup analysis was conducted to compare the grades of nephrotoxicity (grades 1–4). The results showed that DDP-based chemotherapy developed more renal toxicity (OR = 0.40, 95% CI = 0.24–0.68, P = 0.001). Therefore, NDP-based chemotherapy might be an alternative choice for patients with adequate hematopoietic function but do not tolerate DDP-based chemotherapy. However, DDP-based chemotherapy should be considered when patients can tolerate the gastrointestinal side effects and have adequate renal function.

This meta-analysis was a systematic retrieval and review of the medical literature, with comprehensive exploration in subgroup analysis and cumulative analysis. All heterogeneities were insignificant. Both the fixed model and random model were used and the results remained the same. However, our analysis also had some limitations. First, as with any meta-analysis, the results were affected by the quality of the included studies. Second, analyses were based on abstracted data and not on individual patient data (IPD). In general, an IPD-based meta-analysis would give a more robust estimation for the association; therefore, researchers should interpret our results with care, especially for a positive association in subgroup analyses. Third, publication bias is a significant threat to the validity of meta-analysis. Although we detected no evidence of publication bias using graphical and statistical methods, this possibility is difficult to completely rule out. Efficacy was studied as the primary endpoint and toxicities as the secondary endpoint in all 14 studies. We also noted that patients receiving DDP-based chemotherapy developed nausea and vomiting more frequently, which might lead to a deterioration in quality of life (QOL). Given that the primary role of chemotherapy in patients with advanced NSCLC is palliative, the influence on patients’ QOL is an important issue in determining the true value of this therapy. However, none of the 14 trials performed formal QOL assessments. Therefore, further studies will be necessary to assess the differences in QOL between the two regimens. Finally, the current results are mainly based on data from East Asia, and require further confirmation in western countries.

In conclusion, this meta-analysis indicated that DDP-based chemotherapy showed non-inferior antitumor efficacy compared with NDP-based chemotherapy. The toxicity profile might play an important role in the selection of DDP-based or NDP-based regimens. NDP-based regimens might be a better choice for patients unable to tolerate DDP’s toxicities and have adequate hematopoietic function. By contrast, DDP-based regimens are more suitable for patients with adequate renal function and can tolerate gastrointestinal toxicities.

## Methods

### Literature search strategy

An electronic search of the PubMed/Medline, EmBase, Cochrane Library, and China National Knowledge Infrastructure databases was performed. The following keywords were used: “non-small cell lung cancer/Carcinoma, Non-Small-Cell Lung,” “chemotherapy,” and “randomized controlled trial.” To limit publication bias, no language limitation, time limitation, or other restrictions were imposed. Reference lists of original articles, review articles, and the Physician Data Query registry of clinical trials were also examined for additional literature. The last retrieval date was 1 October 2014.

### Selection criteria

The inclusion criteria were as follows: (1) randomized controlled trials aimed to compare the substitution of NDP for DDP in combination chemotherapy as first-line treatment for advanced NSCLC; (2) patients must have pathologically confirmed NSCLC and be in clinical III–IV stage; and (3) whatever drug was combined with NDP or DDP had to be the same cytotoxic agents in both treatment arms. The exclusion criteria were as follows: (1) studies with no data for efficacy and safety, including protocols and phase I clinical trials; (2) studies based on overlapping patients; (3) case reports, abstracts, reviews, conference reports, and experiments.

### Validity assessment

We performed an open assessment of the trials and used the Jadad Scale reported by Jadad *et al.*^28^.

### Data extraction

To avoid bias in the data abstraction process, two authors (YL and SWL) independently extracted data from the trials and compared results. Discrepancies were resolved by third party (HXC) adjudication. Although some papers did not contain all the data, the following information was obtained from each source article as follows: first author, year of publication, number of randomly assigned patients, percentage of male and stage IV, mean age, and chemotherapy regimens. Primary outcomes were overall response rate, OS, and survival probability. Secondary outcomes were specific toxicity data, such as anemia, neutropenia, thrombocytopenia, nausea and vomiting, nephrotoxicity, and neurotoxicity. Toxicity profiles were graded according to the WHO’s criteria or the cooperative groups’ criteria. Figures were electronically digitized, and Kaplan–Meier curves were downloaded by an appropriate software Engauge Digitizer (http://digitizer.sourceforge.net/).

### Statistical analysis

All analyses were performed using the STATA 12.0 package (StataCorp, College Station, TX, USA). HR with 95% confidence interval (CI) was used for OS as demonstrated by Parmar MK *et al.*^29^. For binary data, including overall response, survival probability, and toxicities, the risk ratio (RR) and odds ratio (OR) with 95% CI were used. A Mantel–Haenszel method was used to estimate the summary HR, RR, OR, and their 95% CI. The original values were used for analysis. HR >1 reflects more deaths or progression in the NDP-based arm. RR or OR >1 reflects a favorable outcome in the NDP-based arm for response and survival probability, or an unfavorable outcome for toxicities. A fixed-effects model was used, followed by a random-effects model to confirm all the results. Cumulative meta-analysis was performed to sort out the time-tendency of outcomes, and meta-regression was performed to explain some heterogeneity. Subgroup analyses were conducted by potential confounding factors selected by reviewing the characteristics of included studies. Statistical significance was set at P < 0.05. For each study, the between-study heterogeneity was assessed using χ^2^-based Q statistics and the I^2^ test. Heterogeneity was considered at either P < 0.05 or I^2^ > 50%. The I^2^ index was expressed as a percentage of the proportion of variability of the results because of heterogeneity as opposed to the sampling error. For I^2^ = 0.0%, variability across trials was due to chance rather than heterogeneity. Publication bias was detected by graphical funnel plots. Asymmetry of the funnel plot was tested by Begg’s test and Egger’s test^30^,^31^, and significance was determined at P < 0.05. This article followed the QUORUM and Cochrane Collaboration guidelines for reporting meta-analysis, and agreed with the preferred reporting items for systematic reviews and meta-analyses guidelines^32^.

## Author Contributions

Conceived and designed the experiments: Y.L., J.F.F. and H.X.C. Analyzed the data: Y.L., S.W.L. and S.R.Y. Contributed reagents/materials/analysis tools: Y.L., J.Z.W., J.F.F. and R.M. Wrote the first draft of the manuscript: Y.L., S.R.Y. and J.F.F. Reviewed, edited, and approved the manuscript: Y.L., S.R.Y., S.W.L., R.M., J.Z.W. and J.F.F.

## Additional Information

**How to cite this article**: Liu, Y. *et al.* Comparison of nedaplatin-based versus cisplatin-based chemotherapy for advanced non-small cell lung cancer among East Asian populations: A meta-analysis. *Sci. Rep.* **5**, 10516; doi: 10.1038/srep10516 (2015).

## Figures and Tables

**Table 1:** Basic characteristics of the studies included in this meta-analysis.

**Study**	**Quality (scores)**	**Regimen**	**n**	**Age**	**Male(%)**	**Stage(%)**	**CR + PR**
Furuse *et al.*^14^/1992	3	NDP 90 mg/m^2^d1 + VDS 3mg/m^2^d1,8	68	62	68	65	8
		DDP 90 mg/m^2^d1 + VDS 3 mg/m^2^d1,8	62	62	65	45	9
Xu *et al.*^15^/2002	3	NDP 80–100mg/m^2^d1 + VDS 3 mg/m^2^d1,5,q3w	60	56	70	–	16
		DDP 30mg/m^2^d1–3 + VDS 3 mg/m^2^d1,5,q3w	62	53	84	–	16
Li *et al.*^16^/2006	3	NDP 80mg/m^2^d1 + NVB 25 mg/m^2^d1,8,q4w	25	51	72	–	8
		DDP 80 mg/m^2^d1 + NVB 25 mg/m^2^d1,8,q4w	21				5
Liao *et al.*^17^/2007	3	NDP 100 mg/m^2^d1 + GEM 1000 mg/m^2^d1,8,q3w	24	56	67	63	9
		DDP 80–100 mg/m^2^d1 + GEM 1000 mg/m^2^d2,8,q3w	19				7
Chen *et al.*^18^/2007	3	NDP 30 mg/m^2^d1–3 + PTX 175 mg/m^2^d1,q4w	32	52	66	59	13
		DDP 30 mg/m^2^d1–3 + PTX 175 mg/m^2^d1,q4w	28	56	71	57	13
Li *et al.*^19^/2009	2	NDP 80 mg/m^2^d1 + PTX 175 mg/m^2^d1,q3w	30	47	50	–	11
		DDP 80 mg/m^2^d1 + PTX 175 mg/m^2^d1,q3w	30	47	47	–	10
Lu *et al.*^20^/2009	3	NDP 80 mg/m^2^d1 + NVB 25 mg/m^2^d1,8,q4w	30	54	77	–	10
		DDP 30 mg/m^2^d1–3 + NVB 25 mg/m^2^d1,8,q4w	30				9
Sun *et al.*^21^/2010	2	NDP 100 mg/m^2^d1 + PTX 135 mg/m^2^d1,8,q3w	76	56	66	43	38
		DDP 50 mg/m^2^d2,3 + PTX 135 mg/m^2^d1,8,q3w	74	57	62	46	36
Wang *et al.*^22^/2011	2	NDP 30 mg/m^2^d1–3 + PTX Lip 135 mg/m^2^d1,q4w	37	57	57	–	15
		DDP 30 mg/m^2^d1–3 + PTX Lip 135 mg/m^2^d1,q4w	37				16
Xia *et al.*^23^/2011	3	NDP 80 mg/m^2^d1 + GEM 1000 mg/m^2^d1,8,q4w	126	67	83	24	48
		DDP 75 mg/m^2^d1 + GEM 1000 mg/m^2^d1,8,q4w	120	66	82	26	44
Li *et al.*^24^/2012	3	NDP 80 mg/m^2^d1 + GEM 1250 mg/m^2^d1,8,q3w	29	54	69	59	13
		DDP 75 mg/m^2^d1 + GEM 1250 mg/m^2^d1,8,q3w	27	51	85	63	11
Liang *et al.*^25^/2012	3	NDP 80 mg/m^2^d1 + GEM 1000 mg/m^2^d1,8,q3w	31	69	68	33	12
		DDP 25 mg/m^2^d1–3 + GEM 1000 mg/m^2^d1,8,q3w	29				13
Wang *et al.*^26^/2012	3	NDP 40–50 mg/m^2^d1,8 + TXT 30–35 mg/m^2^d1,8,q3w	41	56	61	54	16
		DDP 25–30 mg/m^2^d1–3 + TXT 75 mg/m^2^d1,q3w	44	54	55	45	17
Qiao *et al.*^27^/2013	2	NDP 75 mg/m^2^d1 + TXT 75 mg/m^2^d1,q3w	25	50	80	28	10
		DDP 75 mg/m^2^d1 + TXT 75 mg/m^2^d1,q3w	25	51	72	44	10

NDP, nedaplatin; DDP, cisplatin; VDS, vindesine; NVB, vinorelbine; GEM, gemcitabine; PTX, paclitaxel; PTX Lip, paclitaxel liposome; TXT, docetaxel; CR, complete response; PR, partial response.

**Table 2:** Outcomes of toxicity meta-analysis.

**Toxicity**	**Trials**	**Heterogeneity P-value**	**Heterogeneity I**^**2**^	**OR (95% CI)**	**P-value**
3–4 Grade anemia	8	0.63	0%	1.01 (0.67–1.53)	0.95
3–4 Grade neutropenia	13	0.95	0%	1.07 (0.81–1.40)	0.65
1–4 Grade neutropenia	11	0.96	0%	1.07 (0.80–1.42)	0.66
3–4 Grade thrombocytopenia	12	0.19	0%	1.68 (1.18–2.40)	0.004
3–4 Grade nausea and vomiting	11	0.48	0%	0.28 (0.20–0.40)	<0.001
3–4 Grade neurotoxicity	4	0.64	0%	0.62 (0.16–2.41)	0.49
3–4 Grade nephrotoxicity	4	0.78	0%	0.31 (0.09–1.06)	0.06
1–4 Grade nephrotoxicity	8	0.93	0%	0.40 (0.24–0.68)	0.001

OR, odds ratio; CI, confidence interval.

**Table 3:** Subgroup analysis of the meta-analysis.

**Outcomes**	**Subgroup**	**No.**	**Effect (95% CI)**	**Estimate for overall effect**	**Heterogeneity**
Overall response	Patient age ≤ 60	11	1.03 (0.86–1.22)	P = 0.76	I^2^ = 0%, P = 1
	Patient age > 60	3	0.97 (0.74–1.28)	P = 0.84	I^2^ = 0%, P = 0.79
	Plus VDS	2	0.95 (0.58–1.56)	P = 0.84	I^2^ = 0%, P = 0.66
	Plus NVB	2	1.20 (0.67–2.16)	P = 0.55	I^2^ = 0%, P = 0.76
	Plus GEM	4	1.02 (0.80–1.30)	P = 0.90	I^2^ = 0%, P = 0.95
	Plus PTX	4	0.99 (0.78–1.25)	P = 0.94	I^2^ = 0%, P = 0.95
	Plus TXT	2	1.01 (0.66–1.53)	P = 0.98	I^2^ = 0%, P = 0.98
	Japan	1	0.81 (0.33–1.97)	P = 0.64	N/A
	China	13	1.02 (0.88–1.18)	P = 0.80	I^2^ = 0%, P = 1
	Overall	14	1.01 (0.87–1.17)	P = 0.89	I^2^ = 0%, P = 1
Overall Survival	Patient age ≤ 60	3	0.82 (0.54–1.22)	P = 0.32	I^2^ = 0%, P = 0.79
	Patient age > 60	2	1.08 (0.88–1.32)	P = 0.45	I^2^ = 0%, P = 0.86
	Plus VDS	1	1.04 (0.65–1.66)	P = 0.87	N/A
	Plus GEM	2	1.08 (0.87–1.34)	P = 0.50	I^2^ = 0%, P = 0.70
	Plus PTX	2	0.79 (0.50–1.25)	P = 0.32	I^2^ = 0%, P = 0.52
	Japan	1	1.04 (0.65–1.66)	P = 0.87	N/A
	China	4	1.02 (0.85–1.22)	P = 0.85	I^2^ = 0%, P = 0.57
	Overall	5	1.02 (0.85–1.22)	P = 0.82	I^2^ = 0%, P = 0.74
0.5-year survival	Patient age ≤ 60	3	0.91 (0.31–2.73)	P = 0.87	I^2^ = 0%, P = 0.87
	Patient age > 60	2	1.31 (0.91–1.89)	P = 0.15	I^2^ = 0%, P = 0.88
	Plus VDS	1	1.26 (0.68–2.33)	P = 0.46	N/A
	Plus GEM	2	1.30 (0.85–2.00)	P = 0.23	I^2^ = 0%, P = 0.69
	Plus PTX	2	0.78 (0.13–4.65)	P = 0.79	I^2^ = 0%, P = 0.62
	Japan	1	1.26 (0.68–2.33)	P = 0.46	N/A
	China	4	1.27 (0.83–1.92)	P = 0.27	I^2^ = 0%, P = 0.88
	Overall	5	1.27 (0.90–1.79)	P = 0.18	I^2^ = 0%, P = 0.95
1-year survival	Patient age ≤ 60	3	0.85 (0.53–1.36)	P = 0.49	I^2^ = 0%, P = 0.92
	Patient age > 60	2	1.10 (0.87–1.38)	P = 0.44	I^2^ = 0%, P = 0.95
	Plus VDS	1	1.08 (0.65–1.79)	P = 0.77	N/A
	Plus GEM	2	1.08 (0.84–1.39)	P = 0.55	I^2^ = 0%, P = 0.57
	Plus PTX	2	0.93 (0.47–1.81)	P = 0.57	I^2^ = 0%, P = 0.69
	Japan	1	1.08 (0.65–1.79)	P = 0.77	N/A
	China	4	1.04 (0.82–1.30)	P = 0.77	I^2^ = 0%, P = 0.78
	Overall	5	1.04 (0.85–1.28)	P = 0.69	I^2^ = 0%, P = 0.90
					

VDS, vindesine; NVB, vinorelbine; GEM, gemcitabine; PTX, paclitaxel; TXT, docetaxel; CI, confidence interval; N/A, Not Applicable.
